# Planar cell polarity genes *frizzled4* and *frizzled6* exert patterning influence on arterial vessel morphogenesis

**DOI:** 10.1371/journal.pone.0171033

**Published:** 2017-03-02

**Authors:** Rene Markovič, Julien Peltan, Marko Gosak, Denis Horvat, Borut Žalik, Benjamin Seguy, Remi Chauvel, Gregoire Malandain, Thierry Couffinhal, Cécile Duplàa, Marko Marhl, Etienne Roux

**Affiliations:** 1 Department of Physics, Faculty of Natural Sciences and Mathematics, University of Maribor, Maribor, Slovenia; 2 Faculty of Education, University of Maribor, Maribor, Slovenia; 3 INSERM, Biology of Cardiovascular Diseases U1034, Pessac, France; 4 Université de Bordeaux, Biology of Cardiovascular Diseases U1034, Pessac, France; 5 Service des Maladies Cardiaques et Vasculaires, Centre Hospitalier Universitaire de Bordeaux, Bordeaux, France; 6 Institute of Physiology, Faculty of Medicine, University of Maribor, Maribor, Slovenia; 7 Faculty of Electrical Engineering and Computer Science, University of Maribor, Maribor, Slovenia; 8 INRIA, Morpheme team, Sophia Antipolis, France; Thomas Jefferson University, UNITED STATES

## Abstract

Quantitative analysis of the vascular network anatomy is critical for the understanding of the vasculature structure and function. In this study, we have combined microcomputed tomography (microCT) and computational analysis to provide quantitative three-dimensional geometrical and topological characterization of the normal kidney vasculature, and to investigate how 2 core genes of the Wnt/planar cell polarity, *Frizzled4* and *Frizzled6*, affect vascular network morphogenesis. Experiments were performed on f*rizzled4* (*Fzd4*^-/-^) and f*rizzled6* (*Fzd6*^-/-^) deleted mice and littermate controls (WT) perfused with a contrast medium after euthanasia and exsanguination. The kidneys were scanned with a high-resolution (16 μm) microCT imaging system, followed by 3D reconstruction of the arterial vasculature. Computational treatment includes decomposition of 3D networks based on Diameter-Defined Strahler Order (DDSO). We have calculated quantitative (i) Global scale parameters, such as the volume of the vasculature and its fractal dimension (ii) Structural parameters depending on the DDSO hierarchical levels such as hierarchical ordering, diameter, length and branching angles of the vessel segments, and (iii) Functional parameters such as estimated resistance to blood flow alongside the vascular tree and average density of terminal arterioles. In normal kidneys, fractal dimension was 2.07±0.11 (n = 7), and was significantly lower in *Fzd4*^-/-^ (1.71±0.04; n = 4), and *Fzd6*^-/-^ (1.54±0.09; n = 3) kidneys. The DDSO number was 5 in WT and *Fzd4*^-/-^, and only 4 in *Fzd6*^-/-^. Scaling characteristics such as diameter and length of vessel segments were altered in mutants, whereas bifurcation angles were not different from WT. *Fzd4* and *Fzd6* deletion increased vessel resistance, calculated using the Hagen-Poiseuille equation, for each DDSO, and decreased the density and the homogeneity of the distal vessel segments. Our results show that our methodology is suitable for 3D quantitative characterization of vascular networks, and that *Fzd4* and *Fzd6* genes have a deep patterning effect on arterial vessel morphogenesis that may determine its functional efficiency.

## Introduction

Precise and comprehensive measurements of vascular network anatomy are crucial steps for the analysis of normal and pathologic vascular networks, and is of paramount importance for the understanding of several aspects of the vasculature structure and function [1]. Microcomputed Tomography (microCT) has emerged in recent years as the preferred modality for vascular studies, because it provides high-resolution three-dimentional (3D) volumetric data suitable for visualization and analysis at the level of an organ or set of tissues [2]. We have previously used this microCT technique specifically to study the arterial network in a hind limb, heart and kidney [3–5]. However, in most of the studies about 3D analysis of a vascular network, including our previous ones, quantitative characterization of the vessel network pattern suffered several limitations. Except for some general parameters, such as vascular volume, most morphometric parameters are computed primarily after dimensional reduction of the 3D micro-CT volumes to 2D sections, and quantification lacks topographical analysis of the vascular network.

Since vascular networks are highly organized hierarchical structures, an accurate quantitative description of the vascular pattern requires topological analysis of these networks. In the present study, using vessels’ segmentation, which has never been tackled by conventional investigation methods, we have developed a complex analysis of the kidney vascular tree according to the diameter-defined Strahler taxonomy of the vessel segments, which provides a topological description of the branching structure of the arterial network. This methodology enabled us to extract key quantitative parameters from the 3D images, such as (i) The fractal dimension, which measures the ability of a self-similar structure to fill the three-dimensional space it occupies, (ii) Structural parameters, such as hierarchical ordering and diameter, length and branching angles of the vessel segments, both at the global scale and, depending on the hierarchical levels of the vessel, and (iii) Functional parameters, such as estimated resistance to blood flow alongside the vascular tree and average density of terminal arterioles. In the present study, we have applied this methodological approach combined with 3D microCT imaging to provide a quantitative geometrical and topological characterization of the normal kidney vasculature, and to investigate how 2 core genes of the Wnt/Planar Cell Polarity (PCP), *Frizzled4* (*Fzd4*) and *Frizzled6* (*Fzd6*), affect vascular morphogenic events.

Wnt/Planar Cell Polarity (PCP) signaling is a non-canonical Wnt/Frizzled pathway critical for the establishment of tissue polarity that shapes the developing invertebrate and vertebrate embryo [6–8]. It has also been shown that the Wnt/ PCP pathway plays a key role in angiogenesis and vascular morphogenesis [9–12]. Frizzled4 (Fzd4) is one of the serpentine receptors of Wnt involved PCP signaling, and its role has been investigated frequently. It has been shown to be required to form and shape precisely the networks of the 3D epithelial tubes of the kidney [13], and several studies have evidenced its role in the regulation of arterial network organization in several organs such as lung, kidney, and retina [5, 9, 14–19]. In a recent study, we have shown that Fzd4 was expressed in arteries but not in veins, and that deletion of *Fzd4* favors the Wnt/PCP pathway and altered the 3D structure of the arterial vasculature of the heart and the kidney [4]. Frizzled6 (Fzd6) is another receptor that binds several Wnt ligands and seems to act via Disheveled-dependent heterotrimetic G protein coupling [20–22]. Fzd6 is a negative regulator of the canonical Wnt/β-catenin pathway [23]. Compared to Fzd4, the Fzd6 receptor has been investigated less. It has been shown to be implicated in several developmental processes, such as tissue polarity signaling in the epithelium and axon growth [24] and hair patterning [25]. In humans, mutations of the *Fzd6* gene have been shown to be linked to developmental defaults such as lip and palate clefts [26], nail dystrophy [27], and neural tube defects [28]. However, its possible role in angiogenesis and vascular patterning is unknown. These results indicate that these PCP genes may play a role in 3D vascular network morphogenesis, but the evaluation of their role on vessel shape and organization requires complete and accurate analysis of a 3D vascular network of a given organ, enabled by the proposed methodology.

## Material and methods

### Animals and ethical statement

This study was conducted in accordance with both institutional guidelines and those in force in the European Community for experimental animal use, and was approved by the local Ethical Committee “Comité d'Ethique de Bordeaux en Expérimentation Animale” (Agreement n°5012043-A “Analysis of the vasculature in small animals by micro scanner”). The mice used were between 10 and 12 weeks old, and were housed 4 to 6 per cage. Food and water were available *ad libitum*, with a 12-hour dark/light cycle. *Frizzled4* and *Frizzled6* deleted mice (*Fzd4*^-/-^ and *Fzd6*^-/-^) were provided by J. Nathans (Johns Hopkins University, Baltimore, MD). *Fzd4*^+/LacZ^ heterozygous knock-in-mice were bred in C57Bl/6 (8 backcross) and CBA (7 backcross) background and then interbred to generate *Fzd4*^-/-^ and control (wild-type) littermate mice. *Fzd6*^-/-^ were maintained on a C57Bl/6 background. *Fzd6*^-/-^ and *Fzd4*^-/-^ mutant embryos were obtained by natural mating. Littermates of each group of transgenic mice were used as control Wild Type (WT) mice.

### Preparation of the animals for microscanner

Two hours before injection of the contrast medium necessary for mCT analysis, the mice received an intraperitoneal injection of a mixture of antiplatelet drugs (acetylsalicylic acid, 75 mg), vasodilators (molsidomin, 1 mg), and anticoagulants (heparin, 300 IU/kg). After euthanasia by intraperitoneal injection of sodium pentobarbital, a median sternotomy was performed avoiding the mammary arteries. Using a binocular microscope, the trunk of the brachiocephalic artery was exposed up to the aortic arch and cannulated using a 27G needle. The vasculature was washed out with an isotonic solution containing 25,000 IU/L heparin once a right draining atriotomy had been done. When the washing solution became clear, preliminary fixing of the tissues was carried out by injecting 1% paraformaldehyde (PFA) for 1 min under a pressure of 1 meter of water. A mixture of 80% Neoprene Latex (Neoprene Latex Dispersion 671 A, Dupont, France) and barium sulphate powdered to 1 03BCm, (3 g/mL, MicrOpaque^®^ oral solution, Guerbet, France) was then injected gradually under a pressure of 1 meter of water. After removal of the material, the animal was put into formic acid for the latex to harden, then, after dissection, the kidneys were fixed overnight in 4% PFA at 4°C. In control experiments (data not shown), we verified that there was no diffusion of the compound out of the vessels, and no vessel trauma was observed. There was no shrinkage of the latex upon fixing and, consequently, no deformation of the vessel, which rendered quantification possible, as demonstrated by the reproducibility of the results. The histological sections showed that most arteries and arterioles down to a diameter of 20 μm were filled with the contrast medium, and the degree of filling was constant from one animal to another.

### Image processing

**Fig 1:**
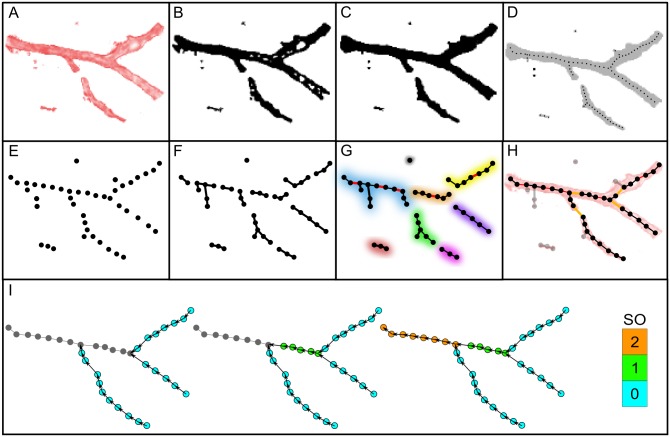
Schematic representation of vessel network extraction and classification algorithm. A: 2D example of an artificial unprocessed vascular segment. Color intensity signifies the HU value of a given voxel. B: Binary image of the vessel after thresholding. Black voxels are foreground voxels, while white voxels characterize the background. C: The application of a morphological closing operator to the binary image. D: Skeletonization of the processed binary image. The skeleton is represented with a thin dotted line. E: Extracted individual centerline points as nodes of a disconnected network. F: Establishing connections between suitable nearest neighbors. G: Connecting close enough nodes with degrees 0 and 1 (red lines, colored segments characterize individual sub-networks). H: Establishing connections between unconnected sub-networks. Light gray nodes and edges signify removed components. I: An illustration of the Strahler classification scheme. The gray nodes have not yet prescribed SO values, whereas other colors of nodes correspond to the SO as given by the color-bar on the right.

The kidney was scanned with a high-resolution micro-CT imaging system (GE eXplore Locus SP), set to a 0.016-mm effective detector pixel size. The apparatus used for imaging the vessels was the eXplore Locus Micro-CT scanner from General Electric Healthcare^®^ with spatial resolutions of 36 to 7 μm, and used with the Scan Control^®^ programs. Our acquisition protocol consisted of 360 views and 4 images per position, with a voxel volume of 16x16x16 μm^3^. To achieve a consistent methodological approach, experimentally acquired voxel images were stored into standard DICOM format, which stands for Digital Imaging and Communications in Medicine. For image processing, we developed our own computational tools in C++ and Python, which are described in detail below and in S1 Appendix. Individual voxels of the 3D image take on the value of the mean attenuation of the scanned material based on the Hounsfield scale (HU). A standard thresholding technique was applied in order to extract most of the embedded vessel structure. For this purpose we have defined a range of HU values between a lower and upper threshold. The upper and lower threshold values have been assessed visually to compensate the differences in perfusion of the vessels with the contrast agent. Voxel values whose HU number was within the specified upper and lower thresholds, were set to 1 (foreground), whilst 0 otherwise (background). However, the resulting binary model may still contain cavities of background voxels. These artifacts emerge mainly due to an inhomogeneous contrast agent filling and can lead to imprecise extraction of the vascular network (see Fig 1A and 1B for illustration). The application of morphological operators has proven to be a useful approach for such issues, also in the filtering of vascular networks [29–31]. Thus, in order to minimalize the effect of these spuriosities, a morphological closing operator was applied to the binary model of the vasculature [32]. In this manner, the spuriosities are filtered out selectively to a large extent, while the general shape of the vascular network was preserved. After applying the closing operator, small connected components which were not a part of the vessel structure were also removed (see Fig 1C). This final representation of the vasculature was then skeletonized as described by Xie *et al*. [33] and illustrated in Fig 1D. The average vessel radiuses were calculated on the basis of the extracted skeleton. Because the cross-sections of the vessels are, in general, not perfect circles (mostly caused by discretization of the vessel), we developed a new procedure for the calculation of the average radius: The neighborhood of each voxel belonging to the skeleton was inspected initially for their local surface voxels (i.e. voxels that are located on the vessel surface). Let *s*_min_ represent the smallest distance between the given skeleton voxel and the surface voxel. Because of the vessel surface irregularity, simply using *s*_min_ to define the radius of the neighborhood would, in most cases, cause an underestimation. To compensate for the vessel’s irregular surface, while still benefiting from the adaptive local neighborhood, 1.5 *s*_min_ was used to define the inspection neighborhood. The nearest distances to the skeleton of the surface voxels contained within the defined neighborhood were then averaged to obtain the average vessel radius for a given skeleton voxel. The skeleton of the vasculature was then stored in an ASCII file, in which each line held the coordinates *x*, *y* and *z* of the skeleton voxel and the average diameter *D* of the vessel at this point. These data were then used to reconstruct the vasculature, define individual vessel segments, and to calculate its structural features, as described below.

### Automated extraction and characterization of the vascular tree

In order to reconstruct the topology of the vascular network in a purely data-driven way, we developed a network-based approach for an automated reconstruction and hierarchical classification of the extracted 3D vascular skeleton. The reconstruction protocol is a two-stage hybrid script written in C++ and Python, using the NetworkX library [34]. In the first stage the centerline and diameter data, extracted during the image segmentation described above, were imported to create individual nodes of the network (see Fig 1E). Each node was defined by a centerline point, which contained additionally the information about the corresponding average diameter. At this point, no edges were established between the nodes, meaning that all nodes had a node degree equal to 0. In parallel, we also computed the average physical distance from each node to its second, fifth and tenth nearest neighbor.

Starting from node *i*, an edge to its suitable nearest neighbor *j* is created if the neighbor *j* has less than 2 existing connections and if the two nodes are not already connected. The node *j* is classified as suitable if the distance between them is less than the average distance between all second-nearest nodes. Applying this procedure to all nodes resulted in a network with node degrees ranging from 0 to 2. At this stage, the network was very segregated and had many unconnected nodes (Fig 1F). To resolve this issue, we focused on all nodes with degrees 0 and 1. We connected each such node if the distance between him and his nearest node was less than the average distance to the fifth-nearest neighbor. An additional criterion, on the basis of which we prevented the creation of loops between adjacent nodes, was the non-existence of a path between the given nodes, which was verified with Dijkstra's algorithm [35]. The edges created additionally in this stage are marked with red in Fig 1G.

Implementing this step resulted in a locally better connected network which could still be segregated in terms of unconnected sub-networks as indicated in Fig 1G with different colors. We therefore interconnected the remaining unconnected components in order to construct a globally connected network. For this purpose, we labeled each node in accordance with the sub-network to which it belonged. All nodes with a degree of 1 in each of the sub-networks were selected and a connection to the nearest node in a different sub-network was established, if the distance between them was less than the average distance to the tenth-nearest node (orange lines in Fig 1H).

Finally, we removed the nodes and segments that were classified as artefacts. All unconnected nodes, i.e. nodes with the degree 0, were removed. Additionally, all nodes with the degree 1 were removed that were separated by not more than one node to the closest bifurcation site. Finally, all smaller and remote unconnected sub-networks were also excluded. All parts of the vasculature that were eliminated on the basis of these criteria, are marked with gray in Fig 1H. As a result, a complete and precise 3D reconstruction of the vessel structure was achieved and purified in a completely data-driven way.

The extracted vascular tree can be regarded as an undirected spatial network whose topological features are identical to the structural characteristics of the exported vascular skeleton. The dominant type of nodes in the constructed network are nodes having 2 edges, i.e. nodes that form the vessel segments. Nodes with a degree of 1 signify terminal parts of the vasculature or the injection inlet site. Nodes with a degree of 3 symbolize bifurcation sites, in which a parent vessel splits into two daughter vessels. To quantify the vascular structures further, we converted the extracted undirected network into a directed one. The introduction of directionality to the vascular network was performed simultaneously with the determination of the Strahler Orders (SO) of the vessel segments, as is shown schematically in Fig 1I. The implemented ordering technique is applied commonly to identify and classify types of streams in tree-like systems [36]. The SO assignment started at the terminal nodes with a degree of 1. From those nodes we followed the path to its geodesically closest bifurcation points. To all embedded nodes on this directed path (oriented from the terminal node to the bifurcation node) an SO of 0 was assigned. Afterwards, starting from each of the selected bifurcation nodes, we followed the path to the closest bifurcation sites, and an SO of 1 was assigned to all nodes on this path. The procedure was repeated until an SO was assigned to all nodes. Moreover, if a parent vessel has multiple daughter vessels with different Strahler Orders, its Strahler Order is 1 order higher than the largest order of the daughter vessels. An unwanted result of this ordering scheme is that the parent vessel segments with a comparable diameter to the connected daughter vessels could be assigned with a different order number. Since the aim of the ordering scheme is to classify vessel segments in accordance to some common feature, Kassab *et al*. introduced the Diameter-Defined Strahler Ordering (DDSO) scheme [37, 38]. Taking the ordinary Strahler Ordering scheme as a starting point, the DDSO of the vessel segments were determined as follows: (i) The mean (*D*_*n*_) and SD (Δ_*n*_) of the vessel segment diameters of order *n* were computed, then (ii) The new order *n* of the *k*-th vessel was assigned such that it satisfied the criterion:

$$\frac{\left(D_{n-1}+\Delta_{n-1}\right)+\left(D_{n}+\Delta_{n}\right)}{2}<D_{n}\leq \frac{\left(D_{n}+\Delta_{n}\right)+\left(D_{n+1}+\Delta_{n+1}\right)}{2}.$$ (1)

These two steps were repeated until the order of the vessel segments remained unaltered. The DDSO ordering scheme produces a hierarchical ranking of vessel segments assigning them an order from 0 for the terminal branches, to a maximal rank for vessels with the highest diameter. The maximal DDSO varies according to the range of vessel diameters and the heterogeneity of their distribution. The whole procedure from the stage of image processing to the segmentation of the vascular networks and the designation of the segments is a multistep process and is presented as a flowchart diagram in the S1 Appendix. Centerline data and the corresponding scaling values are available from the Dryad Digital Repository: http://.doi.org/10.5061/dryad.121c2.

### Metrics for the characterization of the vascular network anatomy

Pure centerline datasets (binary data) of individual samples were used to determine the fractal dimension *D*_f_ and vessel diameter distributions. The fractal dimension was obtained by a standard box-counting algorithm [1, 39]. Briefly, the vasculature was covered with *N*(*x*) equal boxes of sizes *x*, whereby each box contained at least one point of the object. Since the number of non-empty boxes depends on the box size, $N(x)\sim x^{-D_{\text{f}}}$, the fractal dimension can simply be estimated from the best fitting power law function.

Additional quantifications where made on the reconstructed vasculature. Centerline points in the reconstructed vasculature are connected with cylinders. The length of the individual cylinders represents the Euclidean distance between adjacent centerline points. Since the diameters of the two connected centerline points do not necessarily take on the same value, we defined the radius of the cylinder as the average radius of the two centerline points it connected. Next, by following the path between two bifurcation points, or a degree 1 point and a bifurcation point, we defined the vessel segment length and its average diameter. The vessel segment length was computed by summing the Euclidean distances between adjacent nodes contained in a given vessel segment path. The corresponding average diameter was defined respectively as the mean value of the embedded diameter values. The mentioned procedures enabled us to calculate the overall vessel segments`length *I* and volume *V*, which were computed as:

$$l=\sum_{k=1}^{N_{C}}l_{k},$$ (2)

$$l=\sum_{k=1}^{N_{C}}\pi l_{k}{\left(\frac{D_{k}}{2}\right)}^{2},$$ (3)

Where *l*_*k*_ and *D*_*k*_ symbolize the length and the average diameter of the *k*-th vessel segment in the vascular tree and *N*_*C*_ the total number of vessel segments. Further, we computed the absolute and relative distribution of the embedded vessel segment lengths and average diameters, together with the corresponding average values in a given DDSO. The frequency distribution of average diameters *N*(*D*_*k*_) returned the number of vessel segments with an average diameter *D*_*k*_ ∈ [*D*, *D* + *dD*), where *dD* defined the size of the bins. The average *N*(*D*_*k*_) among all samples has been computed and presented for a given phenotype. The corresponding relative distribution of diameters *P*(*D*_*k*_) was defined for every sample by taking its *N*(*D*_*k*_) and normalizing it with the number of vessel segments embedded in it. Again, the average was then presented among all samples of the same phenotype. A similar procedure was used to determine the corresponding distribution *N*(*l*_*k*_) and *P*(*l*_*k*_) regarding the vessel segment lengths. The diameter and length of the vessels are additionally the basic structural features which define its resistance. Following the Hagen-Poiseuille equation, the resistance of a vessel segment is proportional to the length and the viscosity of blood and inversely proportional to the fourth power of the radius [40]. To give an estimation of the resistivity of the vessels in an individual DDSO, we defined *R*_DDSO_ as:

$$R_{\text{DDSO}}=\frac{{\langle l\rangle }_{\text{DDSO}}}{n_{\text{DDSO}}\;{\langle D\rangle }_{\text{DDSO}}^{4}},$$ (4)

Where 〈*l*〉_DDSO_, 〈*D*〉_DDSO_ and *n*_DDSO_ represent the average length, diameter and number of vessel segments in a given DDSO.

For the determination of the bifurcation angles we created position vectors ${\vec{r}}_{i}$ and ${\vec{r}}_{j}$ of the daughter vessels *i* and *j*. The directionality of the position vectors was determined by the directions of the connections between individual nodes. The bifurcation angle *θ* was than computed by means of the scalar product between the two position vectors as follows:

$$\theta ={\text{cos}}^{-1}\left(\frac{{\vec{r}}_{i}\cdot {\vec{r}}_{i}}{\left\|{\vec{r}}_{i}\right\|\left\|{\vec{r}}_{j}\right\|}\right),$$ (5)

Noteworthy, the definition of the bifurcation angle as given in Eq (5) implies that the angles are independent either of the absolute position of the vessel segments or their orientation during the scanning procedure.

### Statistical methods

All statistical analyses were done using OriginPro 8.5 (OriginLab Corporation, Northampton, USA). Statistical comparisons were performed by one-way ANOVA for global scale analysis (total lengh, total volumes and fractal dimension) and two-way ANOVA for DDSO-based analysis (vessel segment length and diameter, bifurcation angles), with the *post-hoc* Tukey test. The homogeneity of bifurcation angles and shortest distance between terminal elements was tested using Levene’s homogeneity test. The correlation between the volume of the kidneys and the vasculature parameters was tested by linear regression analysis. Differences were considered significant when *P*<0.05. Statistical data are given as mean±SD.

## Results

### Global scale analysis of the structure of the arterial vasculature

**Fig 2:**
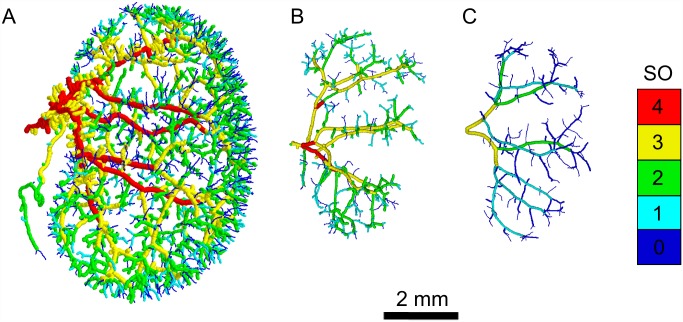
Characteristic kidney vascular networks. Representative processed images of the arterial network of kidneys from WT (A), Fzd4^-/-^ (B), and Fzd6^-/-^ (C) mice. The DDSO of individual vessel segments are color coded, as indicated by the color-bar.

The impact of impaired PCP pathways on vascular network development and vessel-branching morphogenesis was investigated in kidneys from WT (7 samples), *Fzd4*^-/-^, (4 samples) and *Fzd6*^-/-^ (3 samples) mice. Representative extracted vascular networks for WT and both knockouts are displayed in Fig 2. A visual inspection revealed obvious differences between different samples. The vasculatures of both mutant mice (Fig 2B and 2C), in comparison to the WT mice (Fig 2A), were made up of less vessel segments, especially in the terminal part, and occupied a smaller portion of the available space. These features were even more pronounced in the *Fzd6*^-/-^ phenotype. Another feature was the number of DDSO (Fig 2). In WT and *Fzd4*^-/-^ samples 5 DDSO were found (including the terminal vessels), whereas in the *Fzd6*^-/-^ vascular samples, vessels were distributed into 4 DDSO. The smaller number of DDSO in *Fzd6*^-/-^ samples indicates that the vessel diameters were distributed more homogeneously across the whole vasculature and not as much hierarchically as in the WT and *Fzd4*^-/-^ samples.

**Fig 3:**
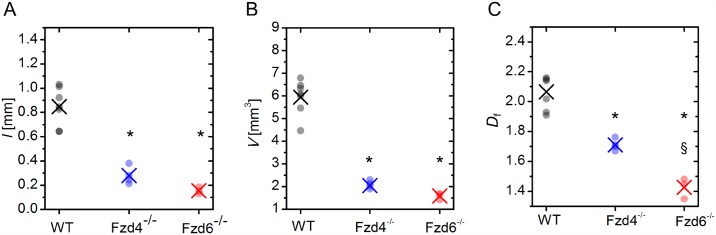
Global structural characteristics of vascular trees. Total overall length *l* of the vasculature (A), overall vessel volume *V* (B) and the fractal dimension *D*_f_ (C). Black: WT, blue: Fzd4^-/-^, red: Fzd6^-/-^. Small dots represent computed values of individual samples in a given phenotype and the crosses signify the corresponding averages. * indicates *P*<0.05 *versus* WT. § indicates *P*<0.05 Fzd4^-/-^ *versus* Fzd6^-/-^.

In order to identify the possible effect of *Fzd4* and *Fzd6* deletion on the size of the organ, we calculated the volumes of the kidneys. Computed kidney volumes were 185.6±5.36 mm^3^ in WT, 135.5±2.42 mm^3^ in *Fzd4*^-/-^, and 1587.7±0.74 mm^3^ in *Fzd6*^-/-^ mice. Statistical analysis showed that mutant kidneys were significantly smaller than WT ones, and, between mutants, *Fzd4*^-/-^ mice had smaller kidneys than *Fzd6*^-/-^ ones. To quantify the global characteristics of the extracted vascular networks, we computed the total length of the vessels *l*, total vessel volume *V* and the fractal dimension *D*_f_. The results are presented in Fig 3. Our calculations have revealed that the average overall length of vessel segments, as well as their volume, were more than two times higher in WT mice than in samples from mutant mice (Fig 3A and 3B), and the difference was statistically significant. Both length and volume were also found to be, on average, a bit higher in *Fzd4*^-/-^ than in *Fzd6*^-/-^ samples, but the differences were not statistically significant. Similarly, the average fractal dimension of WT samples was found to be significantly higher than in the other two phenotypes, and was also significantly higher in frizzled *Fzd4*^-/-^ than in *Fzd6*^-/-^ (Fig 3C). Since the kidneys were found to be significantly smaller in KO versus WT mice, in order to identify the possible influence of the size of the kidneys on the pattern of the vasculature independently from the genotype, we plotted in WT mice the volume and the fractal dimension of the vasculature on the volume of the kidney. The volume of the vasculature was correlated positively with the volume of the organ, but the fractal dimension, which is a scale-free parameter, did not correlate with the volume of the kidney. Individual data and linear regression analysis are given in the S2 Appendix.

### Topological scale analysis of the structure of the arterial vasculature

Analyses of the properties of the vascular trees such as number, diameter and length of vessel segments, as well as branching angles, were performed with regard to the Diameter-Defined Strahler Orders (DDSO) taxonomy.

#### Diameter and length of vessel segments

**Fig 4:**
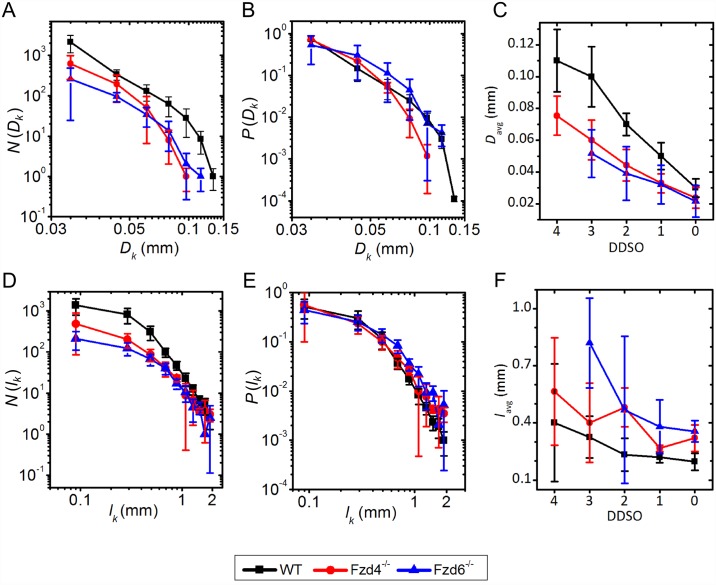
Diameter and length distribution. A, B, C. Log-log plots of absolute (A) and relative (B) diameter distributions, and the average diameters in individual Strahler Orders (DDSO) (C). D, E, F. Log-log plots of absolute (D) and relative (E) vessel segment length distribution and average vessel segment lengths of individual Strahler Orders (F) for WT (black), *Fzd4*^-/-^(blue) and *Fzd6*^-/-^(red) phenotypes. Error bars stand for the SD.

For the analysis, regarding the vessel segment diameters, we performed a standard frequency count, where the number of vessel segments within the diameter range of *D_k_* ∈ [*dD* − *D*, *D* + *dD*] is given by *N*(*D*_*k*_) (see Material and methods). The results are presented in Fig 4A. The number of vessel segments was significantly higher in WT samples than in both mutants, irrespective of the diameter. However, while a higher number of vessels was already expected on the basis of the global scale analysis (Figs 2 and 3), another important difference between phenotypes can be observed in the range of the embedded diameters. In *Fzd4*^-/-^ samples the embedded diameters span over the smallest range, whereas the broadest diameter span of the vessels can be observed in WT samples. To exclude the effect of the variations in the number of vessel segments, we computed additionally the relative diameter distribution, to compare the scaling characteristics directly. The relative diameter distribution *P*(*D*_*k*_) was normalized with the number of vessel segments, and the average diameter with regard to the DDSO classification (see Material and methods). The results presented in Fig 4B indicate that a very similar trend can be observed, except for the largest vessels. Hence, it appears that the relative abundance of vessels with regard to their diameters is comparable between different phenotypes. Lastly, we computed the average diameters for the individual DDSO (Fig 4C). Vascular segments in WT samples had, on average, significantly larger diameters than in *Fzd4*^-/-^ and *Fzd6*^-/-^ ones, but the differences were more pronounced at higher DDSO Orders. In other words, in the terminal vessel segments, the diameters in all phenotypes were in the same range whereas, at higher orders, WT samples exhibited, on average, significantly higher diameters. Comparison between *Fzd4*^-/-^ and *Fzd6*^-/-^ mutants showed that the diameters with regard to the Strahler taxonomy where, on average, significantly lower in *Fzd4*^-/-^ samples, but the difference, though statistically significant, was very small.

Vessel segment length is defined as the physical distance between two bifurcation points. Results showing the absolute and relative distribution of vessel segments lengths and the average vessel segment length in individual DDSO are shown in Fig 4. From the results regarding the absolute frequency distribution of the vessel segment lengths (Fig 4D) we see that the highest number of vessel segments was found in WT samples, whereas the smallest number was observed in the *Fzd6*^-/-^ phenotype. However, the span of the vessel segment lengths appeared not to be affected by the knockout of the receptors. Moreover, the relative distribution of vessel segment lengths (Fig 4E) showed a similar distribution in all three cases. Computation of vessel segment lengths for each individual DDSO is presented in Fig 4F. On average, WT vascular segments were significantly shorter than mutants were and, between mutants, segments were shorter in *Fzd4*^-/-^ samples. These statistical differences were found for terminal segments (DSSO 0 and 1). For intermediate DSSO (2), WT were different from mutants, without difference between mutants. For higher DSSO (3 and 4), no statistical differences were found between samples. It appears that, especially in lower DDSO, there is a higher density of bifurcations, thereby implying a higher level of hierarchy of the vascular networks in WT samples.

#### Bifurcation angles

**Fig 5:**
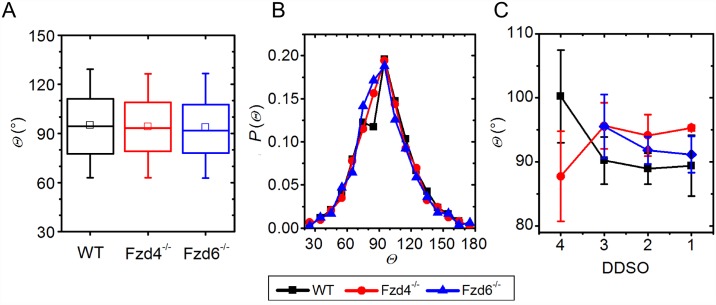
Bifurcation angle distribution. A & B: Bifurcation angles (*Θ*) for individual groups for the whole organ (A) and their frequency distribution (B). C: Average angles per DDSO. In the box chart diagrams in A, the boxes determinate the interval within the 25th and 75th percentiles and whiskers denote the interval within the 5th and 95th percentiles, lines within the boxes indicate the medians, and the small squares stand for the average value. The error bars in panel C stand for the SD. Black: WT, blue: *Fzd4*^*-/-*^, red: *Fzd6*^*-/-*^.

The bifurcation angles at the scale of the whole organ were found to have a similar average value of around 90°, without significant differences between phenotypes, and showed a similar dispersion in all three sub-populations (Fig 5A and 5B). Moreover, the bifurcation angles between different Strahler Orders were also found to be around 90, irrespective of the order and phenotype, whereby the dispersion of calculated angles was more pronounced in higher Strahler Orders (Fig 5B). It appears that, during angiogenesis, the newly formed daughter vessels form a branching angle with the parent vessel of around 90° in WT and mutants. It seems that this mechanism is not affected by modifications in the two core PCP pathway proteins.

### Morphofunctional analysis of the structure of the arterial vasculature

**Fig 6:**
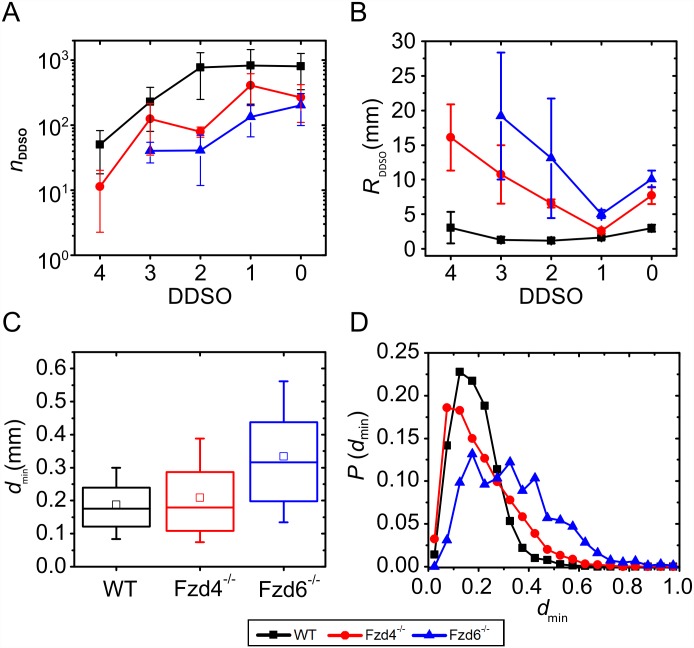
Morphofunctional characteristics of vascular networks. Number of vessel segments (A), estimation of vessel resistance *R*_DDSO_ (B), shortest distance between terminal elements (C) and its frequency distribution (D) for the three sub-populations. In the box chart diagrams (C) the boxes determinate the interval within the 25th and 75th percentiles and whiskers denote the interval within the 5th and 95th percentiles, lines within the boxes indicate the medians, and the small squares stand for the average value. Black: WT, blue: *Fzd4*^-/-^, red: *Fzd6*^-/-^. The error bars in panels A and B stand for the SD.

In order to estimate the functional impact of the structural alterations observed in *Fzd4*^-/-^ and *Fzd6*^-/-^, we have calculated 2 morphofunctional parameters, the resistance of the vessel segments and its distribution along the vascular tree, on which the blood flow rate depends, and the average shortest distance between the distal arterioles, as a surrogate of the density of pre-capillay microcirculation. Results showing the number of vessel segments and the estimated resistance in a given DDSO, together with the average shortest distance between the terminal segments, are shown in Fig 6.

Results (Fig 6A) show, that, in WT samples, most of the vessel segments were assigned a DDSO between 0 and 2 and, for higher values, the number of vessel segments decreased monotonically. The number of vessel segments in *Fzd4*^-/-^ and *Fzd6*^-/-^ was smaller overall. The estimated resistance in individual DDSO was computed by following the Hagen-Poiseuille equation using the number, length and diameter of the vessel segments (see Material and methods, Eq 4). Results are presented in Fig 6B. As expected, the resistance of the WT samples was lower in all DDSO. Indeed, after each bifurcation, the total vessel cross-section through which the blood flows increased. In addition, the vessel segments tended to get shorter, which decreased the resistance additionally. Larger resistances in the two phenotypes were a consequence of the overall smaller diameters and longer vessel segments.

Our methodology did not provide the information on the structure of the capillaries, which are connected to the terminal vessel segments of the extracted structure. To give a rough estimation on the quality of the interweaving network of capillaries, we computed the average shortest distance between the terminal centerline points *d*_min_. Results are presented in Fig 6C and 6D. The shortest distance between terminal parts were 187±89 μm in WT, 209±126 μm in *Fzd4*^-/-^ samples, and 334±166 μm in *Fzd6*^-/-^. The differences were statistically significant in WT *versus Fzd4*^-/-^ and *Fzd6*^-/-^, and in *Fzd4*^-/-^ *versus Fzd6*^-/-^. Moreover, in WT samples, the shortest distance values were distributed much more homogeneously compared to the other two phenotypes (Fig 6D). The variance was significantly lower in WT than in mutants.

## Discussion

In this study, we have used microCT scanning of mouse kidneys, followed by computational skeletonization and topographical analysis to characterize the 3D pattern of the normal arterial vasculature. Applying this methodology to genetically modified mice, we have shown that the cellular arrangements responsible for vascular morphogenesis were coordinated, in part, by two PCP core genes, *Fzd4* and *Fzd6*. MicroCT vascular scanning is the best tool to screen transgenic mice for 3D vascular impairment [2]. However, in most cases, the analysis is limited to the visualization of the vasculature with few, if any, quantitative evaluations of the vascular structure. Skeletonization of mCT is a powerful tool to go beyond the mere visual observation of the vasculature and to provide a detailed study of the changes in structure and their potential effects on hemodynamics. The method was found to generate data with sufficient accuracy and reproducibility to reveal new quantitative information on the pathophysiology of vessel morphogenesis. Algorithms for such reconstructions, based on detailed anatomical data, have been applied to the porcine coronary arterial vasculature [38, 41] and to the cerebral circulation [1, 42, 43]. The accuracy of such procedures depends on the correct identification of the continuity of the vascular tree. During the segmentation, this continuity may be artefactually lost because of inhomogeneous contrast agent filling or pixel discretization of the vessels. Our methodology includes the application of a morphological closing operator that minimalizes these artefactual discontinuities and, hence, allows a more accurate skeletonization of the arterial tree.

For the global analysis of the kidney vasculature, we calculated the total volume as well as the overall length of the vasculature. These global indexes provided quantitative data about the size of the vasculature, and were decreased by both *Fzd4* and *Fzd6* deletion compared with WT mice. Additionally, we calculated the fractal dimension of the vasculature. Fractal analysis is a very interesting tool to investigate a large variety of self-similar biological structures [39, 44], including the vasculature [45, 46]. However, to the best of our knowledge, only two studies, published around 20 years ago, have calculated the fractal dimension of the kidney vasculature [47, 48]. Cross *et al*. have calculated the fractal dimension of human kidney vasculature from angiograms, hence two-dimensional analysis, with a mean fractal dimension of 1.61 [47]. Gil-Gracia *et al*. have used a simplified arterial tree reconstructed from dog kidney vasculature obtained by plastic injection followed by dissolution of organic tissues, and calculated a fractal dimension of 1.94. In this study, we provide a more precise 3D calculation of the fractal dimension of kidney vasculature of normal mice, 2.07, i.e., between 2 and 3. Kidneys from *Fzd4*^-/-^ and *Fzd6*^-/-^ mice have a lower fractal dimension. Additionally, the fractal dimension is even lower in *Fzd6*^-/-^ animals compared to *Fzd4*^-/-^ and WT ones, as is the number of DDSO vessel generations, indicating that *Fzd6* has a greater impact than *Fzd4* on the vascular pattern. Hence, on a global scale, *Fzd4*^-/-^ and *Fzd6*^-/-^ phenotype samples were found to develop smaller kidneys with a shorter overall vessel span. However, their structures were not only a downsized version of the WT vasculature, but exhibited a lower degree of complexity, as indicated by the smaller values of fractal dimension. It seems that the mechanism which governs the development of the vascular structure is affected in a non-trivial way in *Fzd4*^-/-^ and *Fzd6*^-/-^ phenotype samples, with a greater impact of *Fzd6*.

Geometrical analysis based on DDSO provides a more detailed information on the hierarchical organization of the vasculature. It showed that WT kidneys have a broader diameter span and lower average diameters`values in individual DDSO than *Fzd4*^-/-^ and *Fzd6*^-/-^ ones. Vessels with a larger diameter, which were present in the WT samples, were absent in both mutants. The lack of the largest vessels in both mutants may be related with the smaller size of the organ. The distribution of vessel segment length was comparable in WT, *Fzd4*^-/-^ and *Fzd6*^-/-^ mice. However, WT kidney samples have one order of magnitude more vessels in the terminal part and keep a high number of vessel segments in lower DDSO. In *Fzd4*^-/-^ and *Fzd6*^-/-^ kidney samples, the number of terminal segments was lower and their length higher compared to WT, but the 2 deletions did not have the same effect. Terminal segments were shorter in *Fzd4*^-/-^ kidneys, and less numerous in *Fzd6*^-/-^ ones. However, global bifurcation angles and bifurcation angles in individual DDSO showed no significant differences and were found to be around 90°. Taken together, these results indicated that *Fzd4* and *Fzd6* genes contribute differentially to determine the number, diameter and length of the vessel segments, but not the branching pattern of the vessels.

These structural changes may affect the efficiency of the vasculature in terms of blood flow and tissue perfusion. Blood convection through the vessels depends on the resistance of the vascular tree to fluid flow. Indeed, the estimated resistance of WT samples in individual DDSO is smaller compared to *Fzd4*^-/-^ and *Fzd6*^-/-^ kidney samples, suggesting a less efficient vasculature in mutants. We also computed the average shortest distance between the terminal parts of the arterial tree. Their diameter, around 20 μm, lies in the range of that of rat and rabbit renal afferent arterioles [49, 50]. The average shortest distance was found to be a bit less than 200 μm in WT mice. Morphometrical analysis of 2D immunohistochemical preparations from rat kidneys showed that the renal capillary density was around 700/mm^2^ [51]. Assuming a homogenous distribution of the capillaries, this value corresponds to an average distance between 2 adjacent capillaries around 35–40 μm. This suggests that the terminal segments of our study were pre-capillary distal arterioles. The fact that the shortest distance was higher and distributed more heterogeneously in mutants, and particularly in *Fzd6*^-/-^, than in WT kidney samples, shows that deletion of *Fzd4* and *Fzd6* alters the density and the homogeneity of the arterial microcirculation. Taken together, these data suggest that *Fzd4* and *Fzd6* gene expression have critical consequences on the functional efficiency of the vasculature, both in terms of resistance to blood flow convection through the arterial tree, and density and homonegeity of distal arterioles. These morphofunctional data can be considered as relevant indicative parameters of the functional capacities of the vasculature, but it should be noted that they are computed values on simplified hypotheses and not direct measurements.

Fzd4 and Fzd6 deletions not only altered the pattern of the vasculature, but the whole organ, since mutant kidneys were significantly smaller than control ones and, between mutants, *Fzd4*^*-/-*^ had smaller kidneys than *Fzd6*^*-/-*^ mice. Size difference between kidneys may explain some differences in the structure of the vasculature such as its volume, but not its fractal dimension, and the effect of *Fzd4* and *Fzd6* deletions cannot be explained as a mere consequence of the difference in size of the kidneys. For example, the fractal dimension and the number of DDSO were lower in *Fzd4*^*-/-*^ than in *Fzd6*^*-/-*^, whereas the volume of the kidney was higher. Also, if the difference in the vasculature between WT and KO kidneys was only due to the smaller size of KO kidneys, one would expect that the average shortest distance between terminal segments would be smaller (due to the scale reduction), without influence on its heterogeneity, whereas what was observed was just the opposite.

Hence, our results show that *Fzd4* and *Fzd6* have patterning effects on the kidney vasculature, but how these gene products exert these effects remains an open question, since the deletions of these two PCP genes was not tissue-specific. On the one hand, Wnt/PCP genes such as *Fzd4* have been shown to determine the 3D development of the epithelial tubes of the kidney [13], so the effects of *Fzd4* and *Fzd6* may be indirect consequences of the direct developmental role of these genes in renal epithelial cells. On the other hand, recent studies have shown that non-canonical Wnt signaling exerts a direct modeling role in endothelial cells during angiogenesis [5, 52–54], so that the observed effects may be a direct consequence of *Fzd4* and *Fzd6* pathways in endothelial cells. Further studies are required to identify the underlying mechanisms of the observed vascular patterning effects of *Fzd4* and *Fzd6*.

In conclusion, we proposed a methodology based microCT scanning followed by 3D computational segmentation of mouse arterial kidney vasculature that determines quantitative parameters that describe the general pattern of the vasculature, such as overall vessel length and volume and fractal dimension, as well as its scaling characteristics, such as vascular segment numbers, diameters, lengths and angles depending on the vessel hierarchical structure and, additionally, provide indexes of morphofunctional properties. This methodology was found suitable for quantitative comparisons of vascular networks between different conditions. Applying this methodology to genetically modified mice, we have shown that the core PCP genes *Fzd4* and *Fzd6* have deep specific patterning effect on arterial vessel morphogenesis that may determine its functional efficiency.

## Supporting information

S1 Appendix Vasculature segmentation and reconstruction workflow. The flowchart shows the sequential order in which individual subroutines are arranged in our computational protocol in order to segment out the vascular structure and to reconstruct it. Used abbreviation are SO for the Strahler order, SO_*p*_ for the parent vessel Strahler order, SO_*d*_ for the daughter vessel Strahler order, *D*_*n*_ for the mean vessel segment diameter belonging to the *n*-th order, <nn2> for the average physical distance from each node to its second nearest neighbor, <nn5> for the average physical distance from each node to its fifth nearest neighbor and <nn10> for the average physical distance from each node to its tenth nearest neighbor.
(PDF)

S2 Appendix Global scale parameter analysis. (PDF)
