# Supplementary material for: Planar cell polarity genes frizzled4 and frizzled6 exert patterning influence on arterial vessel morphogenesis
Source: PLoS One. 2017 Mar 2;12(3):e0171033. doi: 10.1371/journal.pone.0171033 (PMC5333836; doi:10.1371/journal.pone.0171033)
Supplement: S1 Appendix — The flowchart shows the sequential order in which individual subroutines are arranged in our computational protocol in order to segment out the vascular structure and to reconstruct it. Used abbreviation are SO for the Strahler order, SOp for the parent vessel Strahler order, SOd for the daughter vessel Strahler order, Dn for the mean vessel segment diameter belonging to the n-th order, for the average physical distance from each node to its second nearest neighbor, for the average physical distance from each node to its fifth nearest neighbor and for the average physical distance from each node to its tenth nearest neighbor. (PDF) [file pone.0171033.s001.pdf]

S1 appendix: Vasculature segmentation and reconstruction workflow.

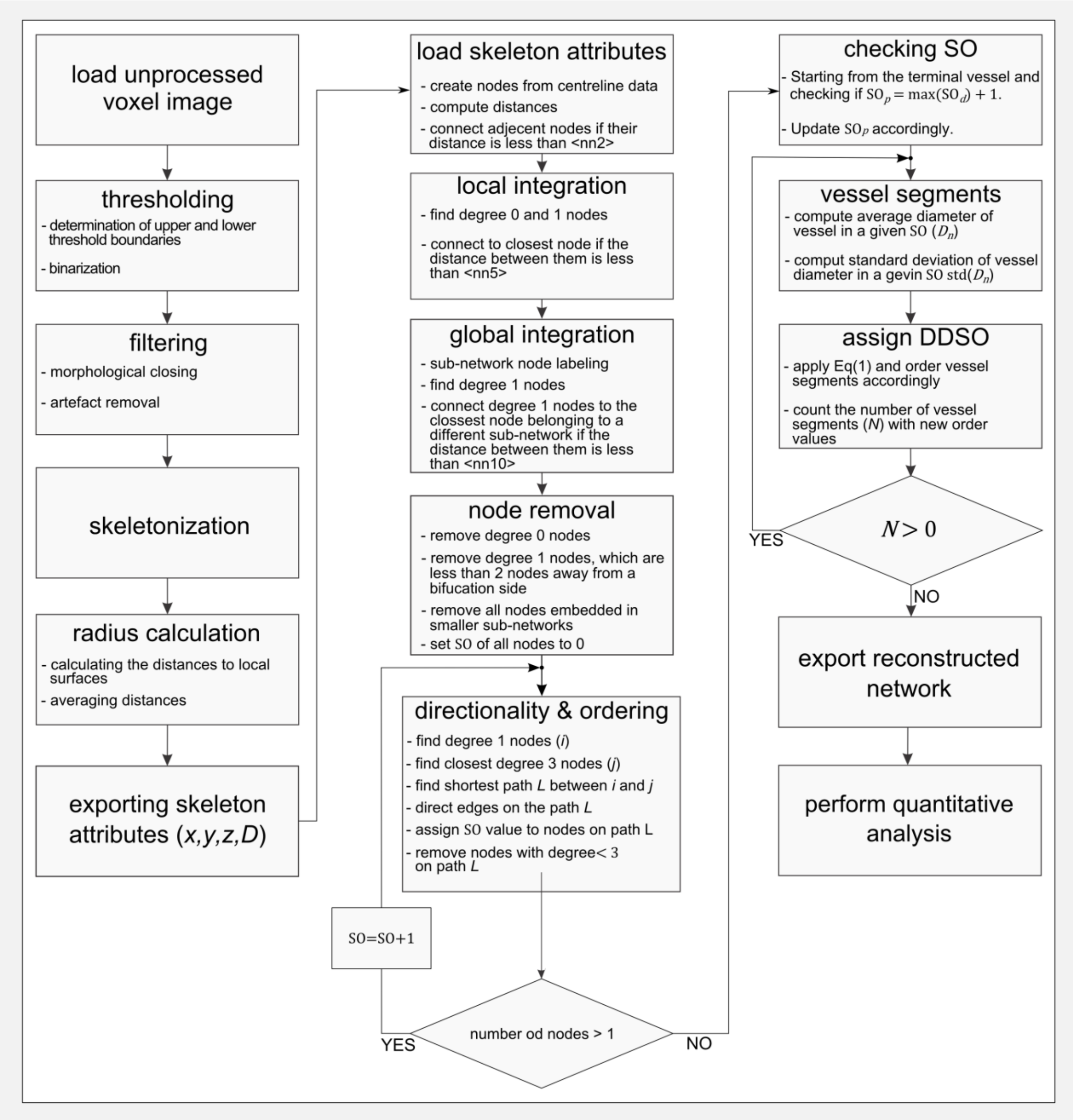

The flowchart shows the sequential order in which individual subroutines are arranged in our computational protocol in order to segment out the vascular structure and to reconstruct it. Used abbreviations are SO for the Strahler Order, SO_p for the parent vessel Strahler Order, SO_d for the daughter vessel Strahler Order, D_n for the mean vessel segment diameter belonging to the n-th order, <nn2> for the average physical distance from each node to its second nearest neighbor, <nn5> for the average physical distance from each node to its fifth nearest neighbor and <nn10> for the average physical distance from each node to its tenth nearest neighbor.
