# Supplementary material for: Planar cell polarity genes frizzled4 and frizzled6 exert patterning influence on arterial vessel morphogenesis
Source: PLoS One. 2017 Mar 2;12(3):e0171033. doi: 10.1371/journal.pone.0171033 (PMC5333836; doi:10.1371/journal.pone.0171033)
Supplement: S2 Appendix — (PDF) [file pone.0171033.s002.pdf]

S2 appendix: Global scale parameter analysis

Calculation of global scale parameters

The following global scale parameters have been calculated: The fractal dimension (D_f), the volume of the vasculature (V), the volume of the kidney V_o and the ratio of the vasculature volume on the organ volume (V/Vo). For the calculation of the volume of the vasculature, we have compared the results obtained with our own algorithms with that obtained using the SourceForge open source MicroView 2.5.0 Software (Parallax Innovations Inc., Ilderton, Canada). Results are given in Table A. Comparison of V values obtained with our algorithm and MicroView® was performed by paired Student *t* test, and the difference was not statistically significant (P>0.05).

Table A. Fractal dimension (D_f), volume of the vasculature (V), volume of the kidney V_o and (V/Vo ratio in WT, *Fzd4*- and *Fzd6*-deleted kidneys.

sample	D_f	V_o	V	V (Microview)	V/V_o
WT 1	2.02	189.39	6.8	6.98	0.0359
WT 2	2.16	192.42	6.47	6.62	0.0336
WT 3	1.91	160.2	3.08	3.26	0.0192
WT 4	1.93	183.92	4.89	4.54	0.0266
WT 5	2.15	174.54	6.09	6.09	0.0349
WT 6	2.15	198.59	6.34	6.5	0.0319
WT 7	2.14	199.91	6.01	6.03	0.0301
Fzd4$^{-/-}$ 1	1.76	130.54	2.49	2.46	0.0191
Fzd4$^{-/-}$ 2	1.7	140.17	1.7	1.69	0.0121
Fzd4$^{-/-}$ 3	1.67	132.15	1.34	1.37	0.0101
Fzd4$^{-/-}$ 4	1.71	139.07	1.5	1.53	0.0108
Fzd6$^{-/-}$ 1	1.48	159.01	1.45	1.41	0.0091
Fzd6$^{-/-}$ 2	1.35	157.74	1.94	1.92	0.0123
Fzd6$^{-/-}$ 3	1.45	156.44	1.62	1.66	0.0104
mean±SD WT	2.07±0.11	185.57±14.17	5.67±1.29	5.72±1.33	0.0303±0.0058
mean±SD *Fzd4*$^{-/-}$	1.71±0.04	134.29±4.84	1.84±0.51	1.84±0.48	0.0130±0.0041
mean±SD *Fzd6*$^{-/-}$	1.4±0.07	157.73±1.29	1.67±0.25	1.67±0.26	0.0106±0.0016

Effect of size on global scale parameters in Wild Type mice

In order to investigate the possible effect of the size of the kidneys on the pattern of the arterial vasculature independently from any morphogenetic impairment, we have plotted individual values of the fractal dimension (D_f), the volume of the vasculature (V), and the ratio of the vasculature volume on the organ volume (V/V_o) against the volume of the kidney V_o. Correlations were tested by linear regression analysis and considered statistically significant when P<0.05. Results are given in Figure A.

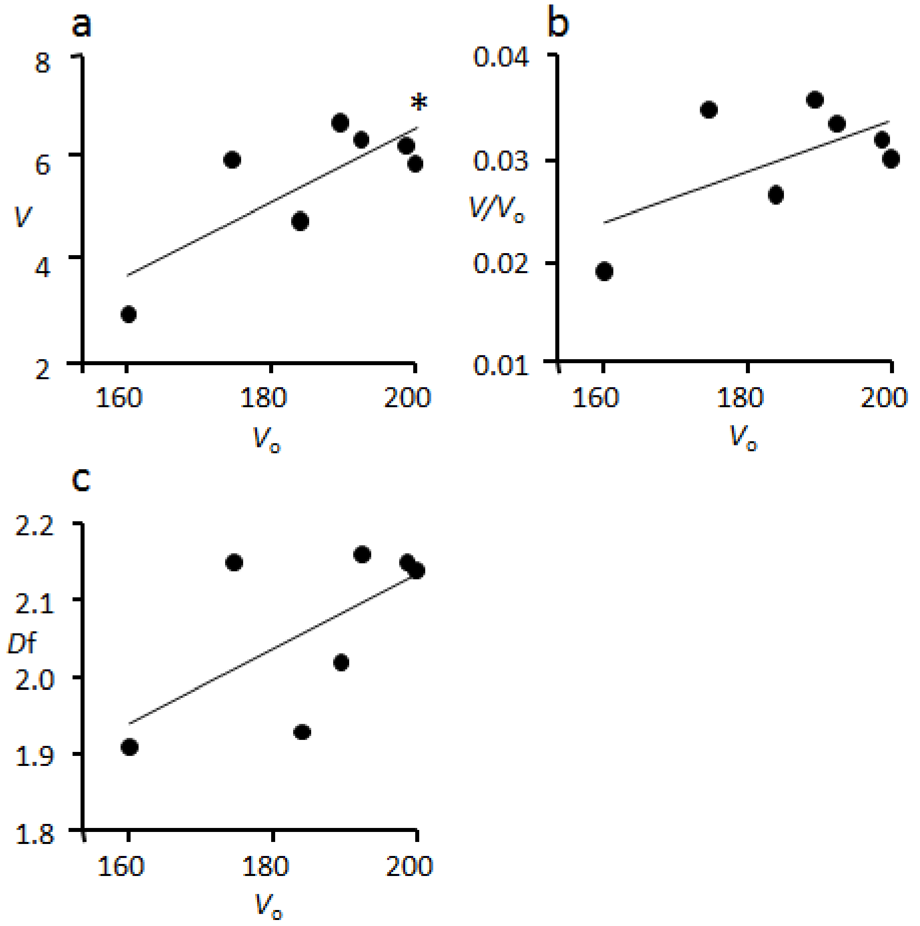

Figure A. Volume of the vasculature *V* (a), ratio of the vasculature volume on the organ volume V/V_o (b) and fractal dimension D_f (c), plotted against the volume of the kidney V_o in WT mice. *The slope of the curve is significantly different from zero. In normal (WT) mice, the volume of the vasculature *V* was correlated positively with the volume of the organ V_o, but the fractal dimension, which is a scale-free parameter, and the V/V_o ratio did not correlate with V_o.
